# Supplementary figures and images for: Molecular mechanism and structure-guided humanization of a broadly neutralizing antibody against SFTSV
Source: PLoS Pathog. 2024 Sep 25;20(9):e1012550. doi: 10.1371/journal.ppat.1012550 (PMC11423973; doi:10.1371/journal.ppat.1012550)

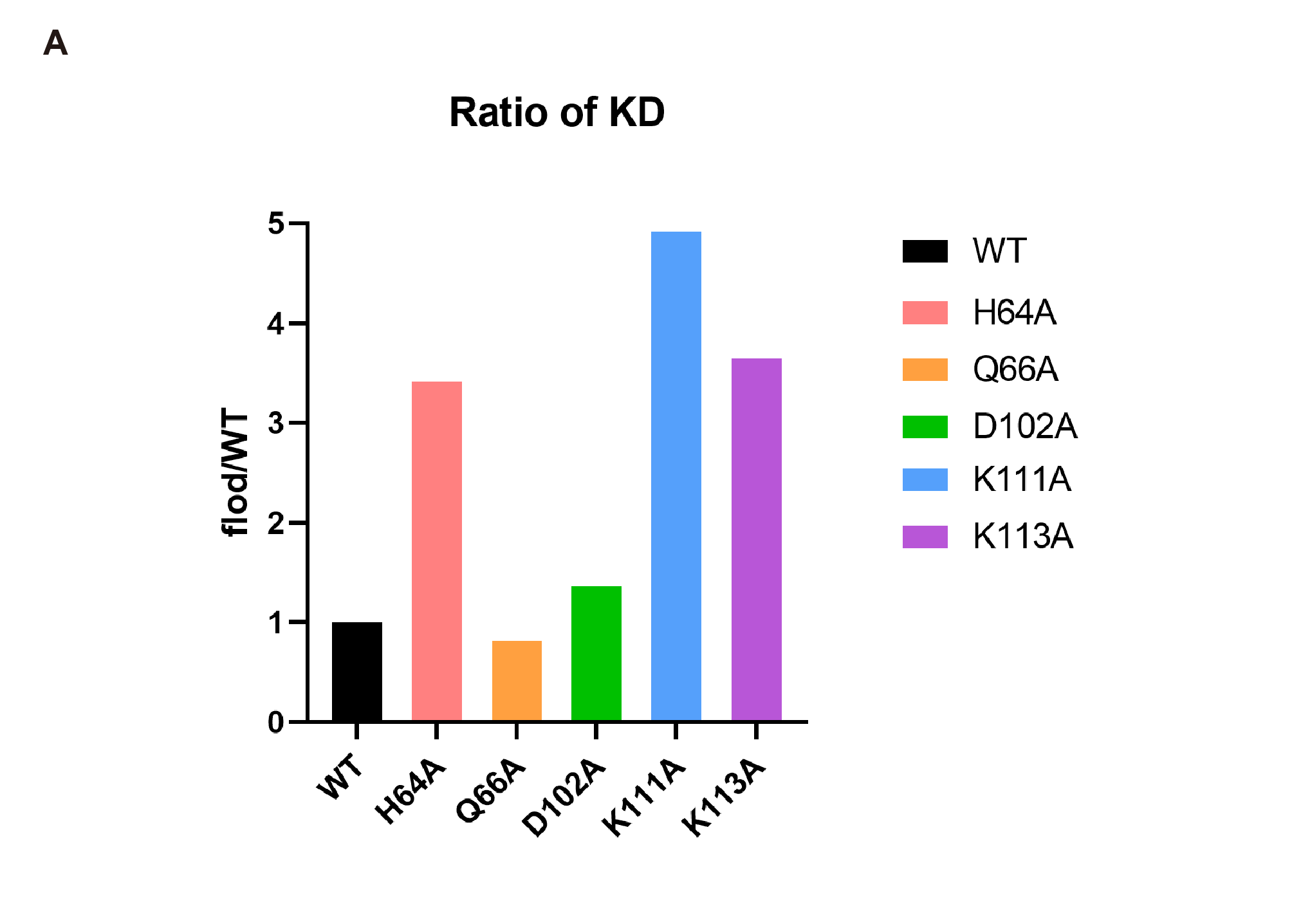

Supplement: S1 Fig — (TIF) [file ppat.1012550.s001.tif]

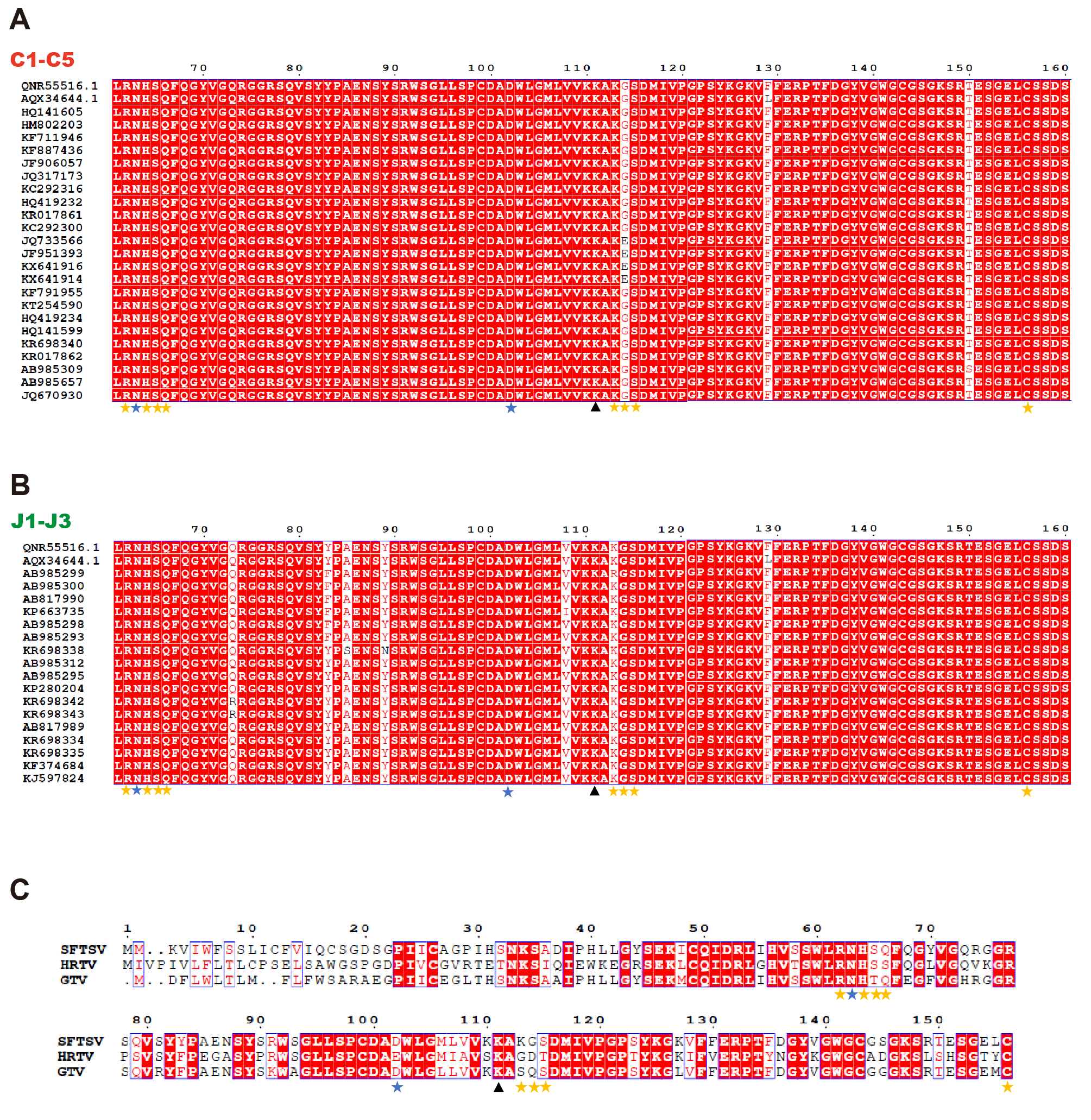

Supplement: S2 Fig — The numbers preceding the sequences are the GeneBank IDs of SFTSV strains. QNR55516.1 and AQX34644.1 are the SFTSV strains used for structural and neutralization analyses in this study, both belonging to the C3 genotype. (A) Chinese lineage SFTSV strains. C1 genotype: HQ141605, HM802203; C2 genotype: KF711946, KF887436, JF906057, JQ317173, KC292316, HQ419232, KR017861; C3 genotype: KC292300, JQ733566, JF951393, KX641916, KX641914; C4 genotype: KF791955, KT254590, HQ419234, HQ141599, KR698340, KR017862; C5 genotype: AB985309, AB985657, JQ670930. (B) Japanese lineage SFTSV strains. J1 genotype: AB985299, AB985300, AB817990, KP663735, AB985298, AB985293; J2 genotype: KR698338, AB985312, AB985295, KP280204, KR698342, KR698343; J3 genotype: AB817989, KR698334, KR698335, KF374684, KJ597824. (C) The sequence alignment of SFTSV with HRTV (GeneBank: AIF75092.1) and GTV (GeneBank: ALQ33264.1). The key residues interacting with the heavy chain of mAb 40C10 are highlighted with blue pentagram, and those interacting with the light chain of mAb 40C10 are marked with yellow pentagram. The residue interacting with both the heavy and light chains of mAb 40C10 are marked with black triangle. (TIF) [file ppat.1012550.s002.tif]

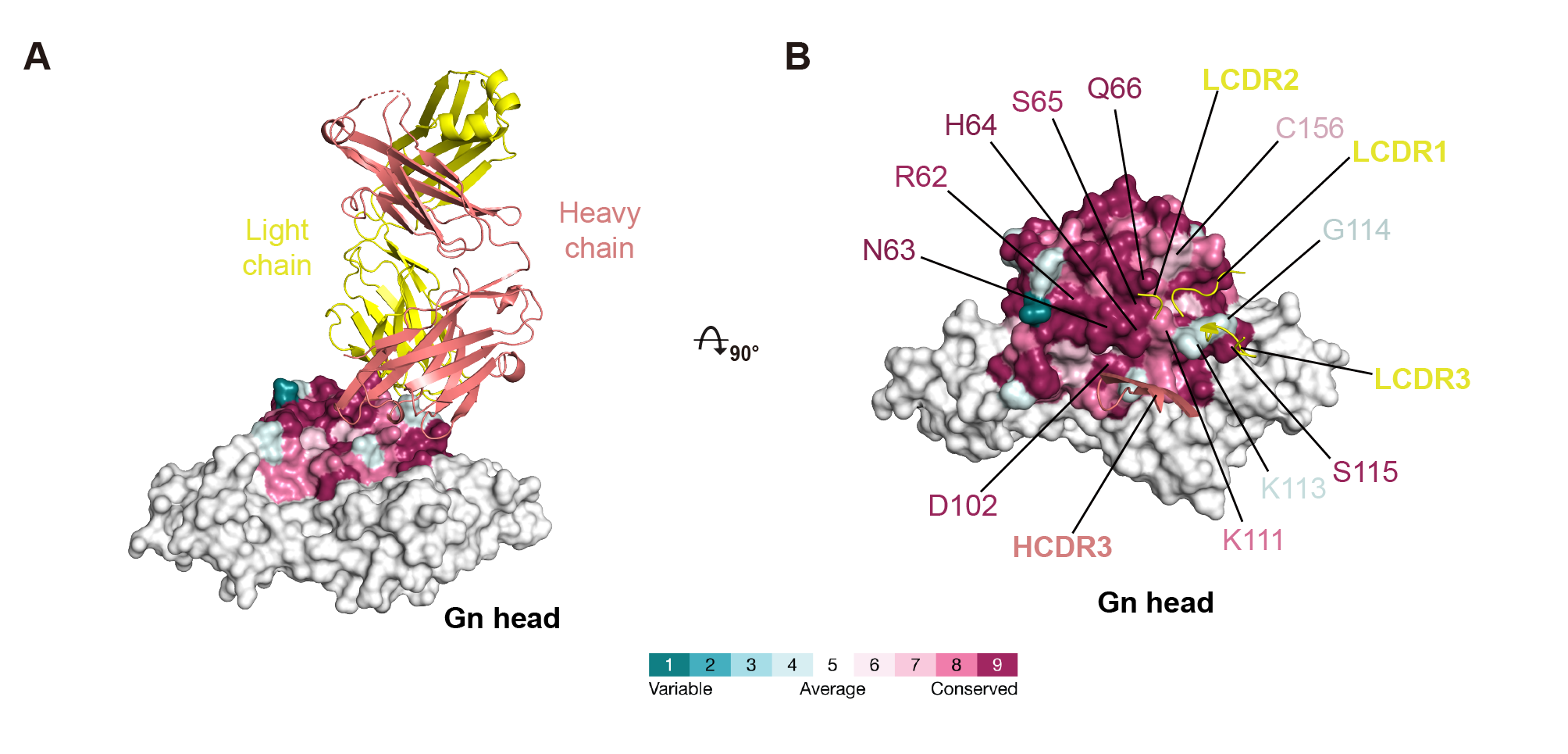

Supplement: S3 Fig — (A and B) The SFTSV Gn head is shown as surface. Domain I of the SFTSV Gn head is colored by sequence variation using the ConSurf server [50,51], while domains II and III are colored gray. The mAb 40C10 heavy and light chains are colored salmon and yellow, respectively. (B) The binding region of mAb 40C10 on the SFTSV Gn head is indicated. The CDR loops of mAb 40C10 colored as described above. (TIF) [file ppat.1012550.s003.tif]

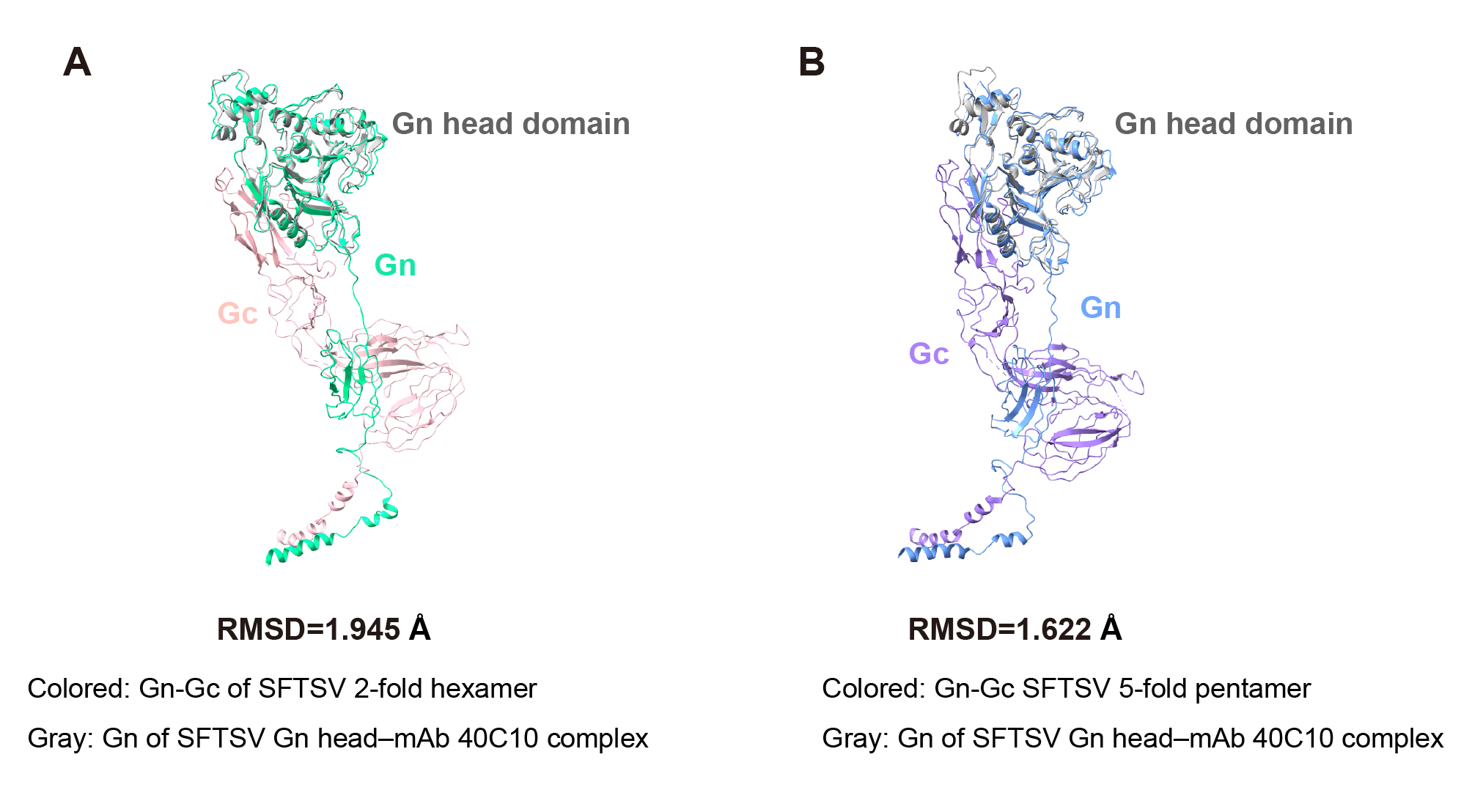

Supplement: S4 Fig — (A and B) The Gn in the SFTSV Gn head–mAb 40C10 complex crystal structure is shown in gray. (A) The Gn-Gc of SFTSV 2-fold hexamer (PDB: 7X6W) is displayed in color. (B) The Gn-Gc of SFTSV 5-fold pentamer (PDB: 7X72) is shown in color. (TIF) [file ppat.1012550.s004.tif]

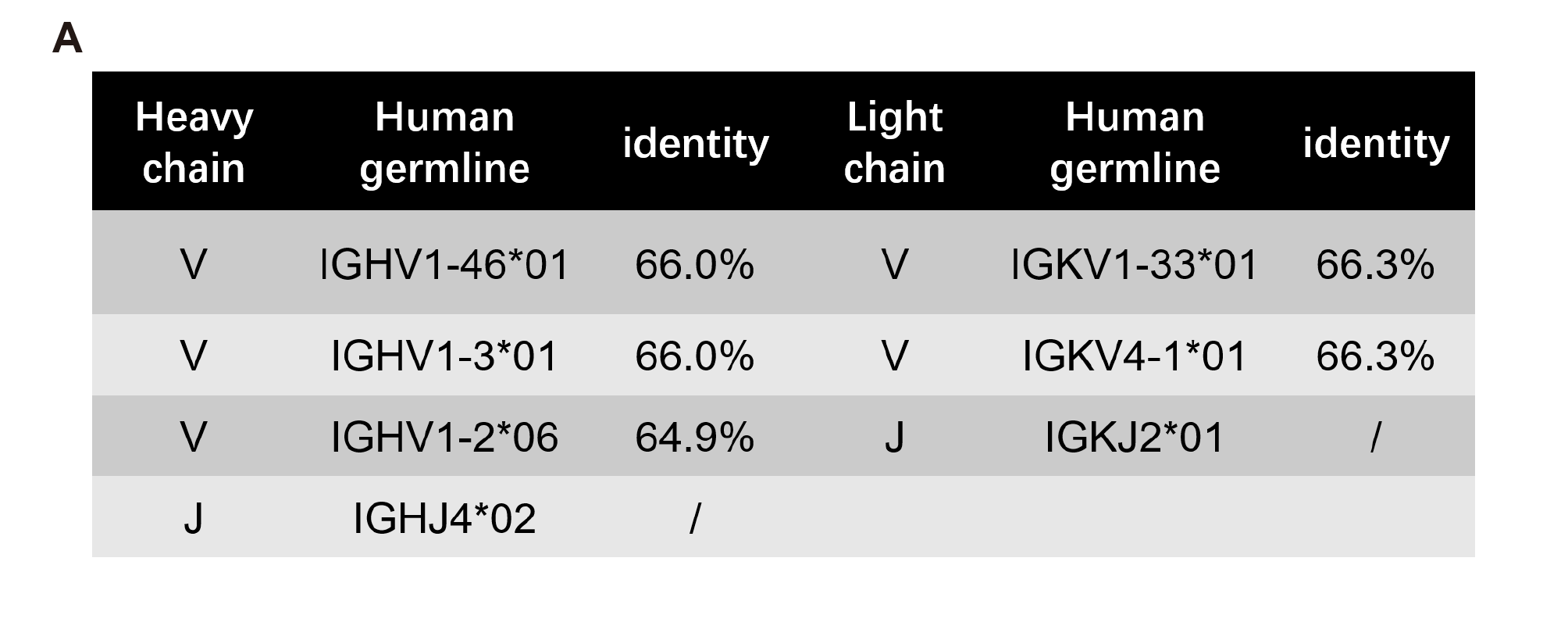

Supplement: S5 Fig — (TIF) [file ppat.1012550.s005.tif]

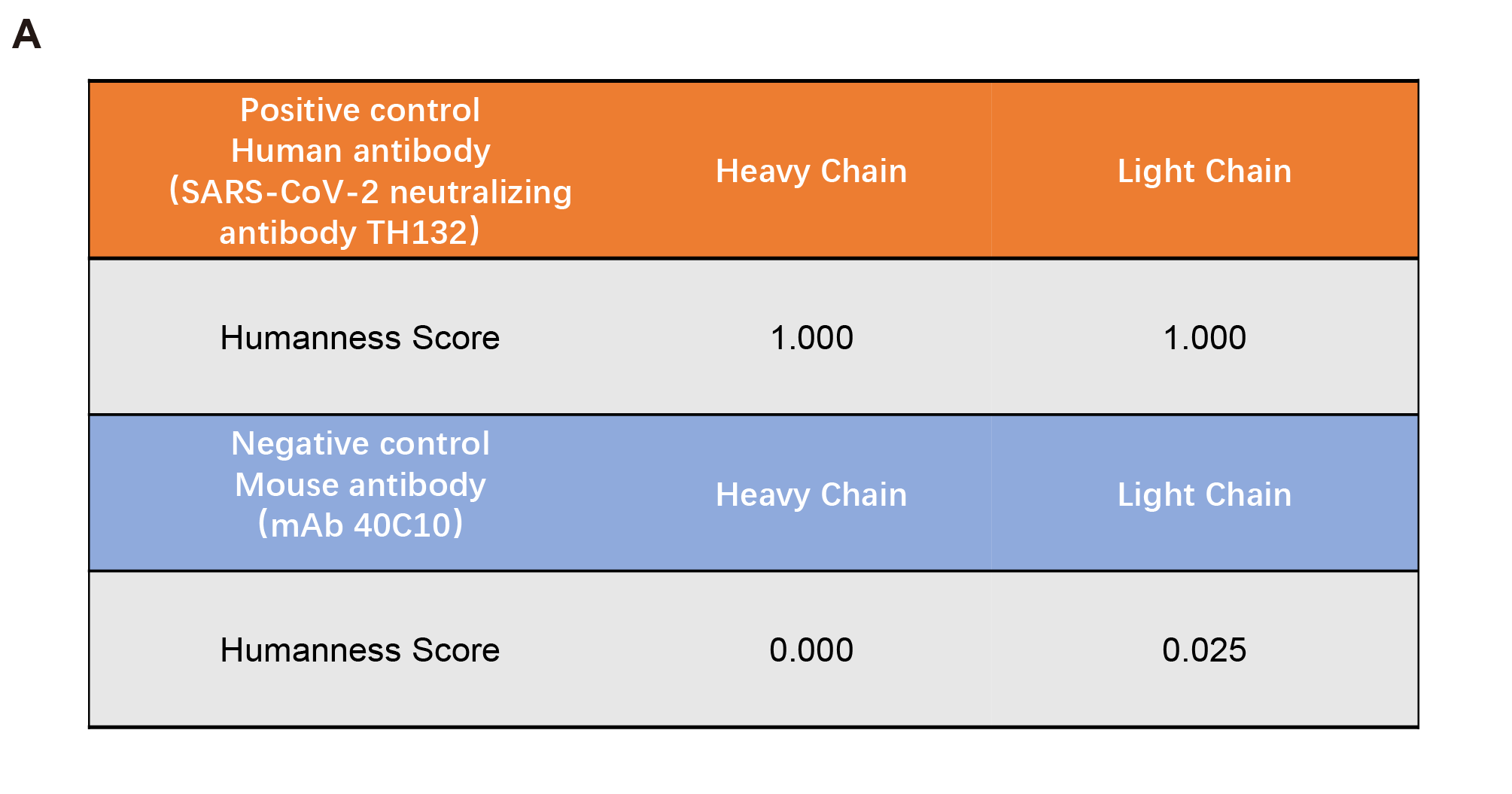

Supplement: S6 Fig — (A) The human antibody (SARS-CoV-2 neutralizing antibody TH132) [52] and the mouse antibody (mAb 40C10) were scored for their degree of humanization using the “Hu-mAb” website. The human antibody serves as a positive control, while the mouse antibody serves as a negative control for humanization scoring. (TIF) [file ppat.1012550.s006.tif]

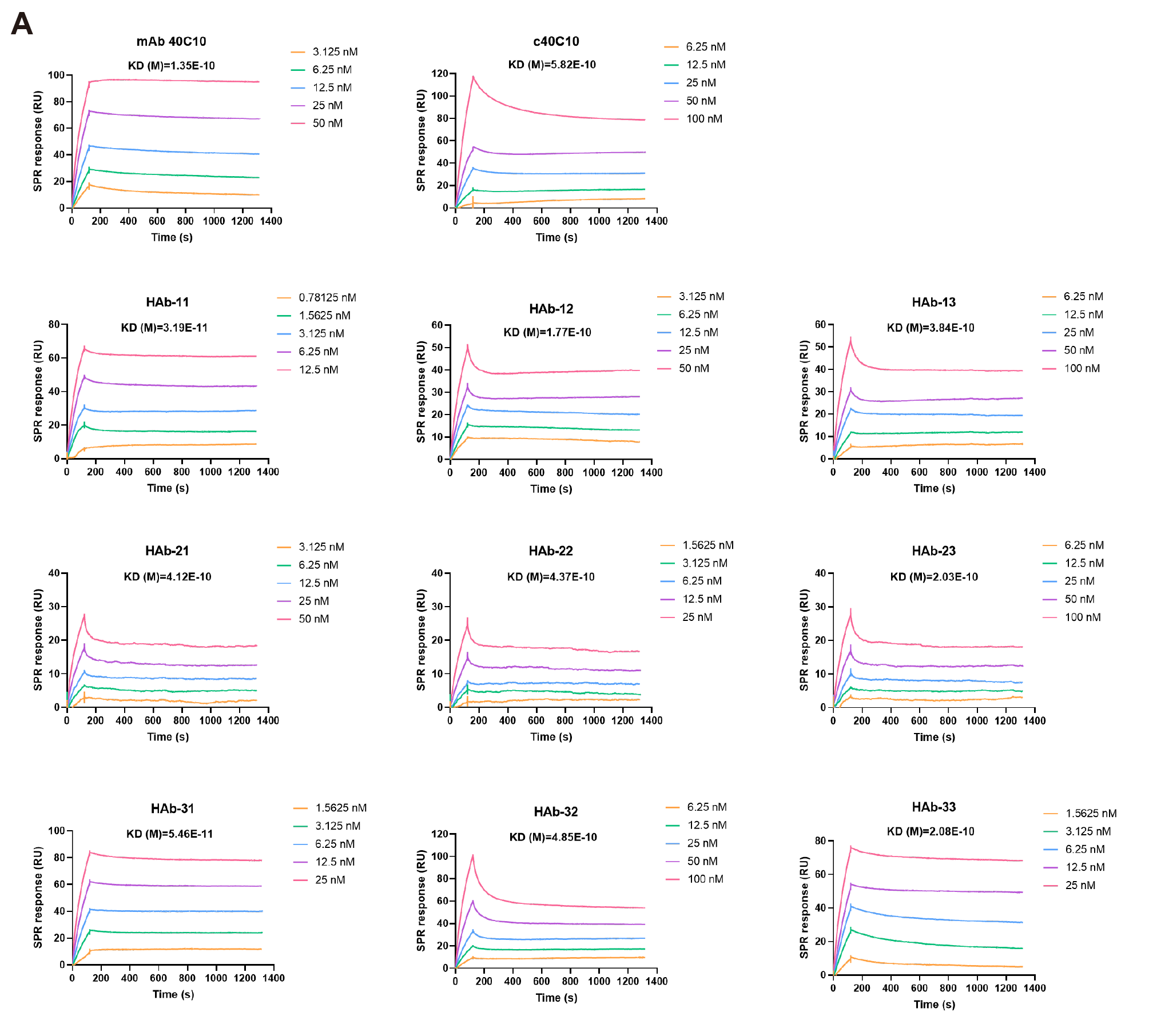

Supplement: S7 Fig — (TIF) [file ppat.1012550.s007.tif]

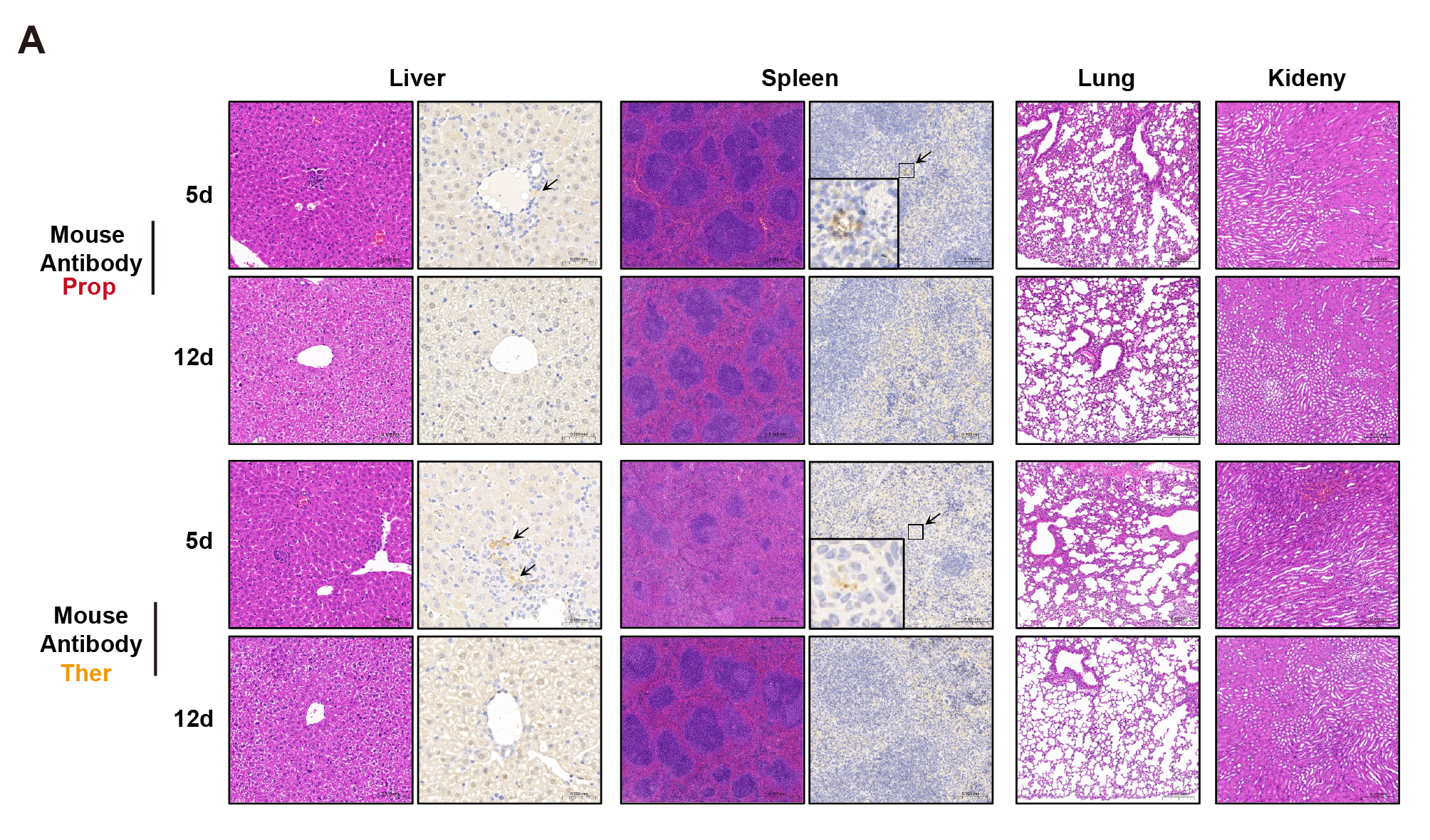

Supplement: S8 Fig — (TIF) [file ppat.1012550.s008.tif]

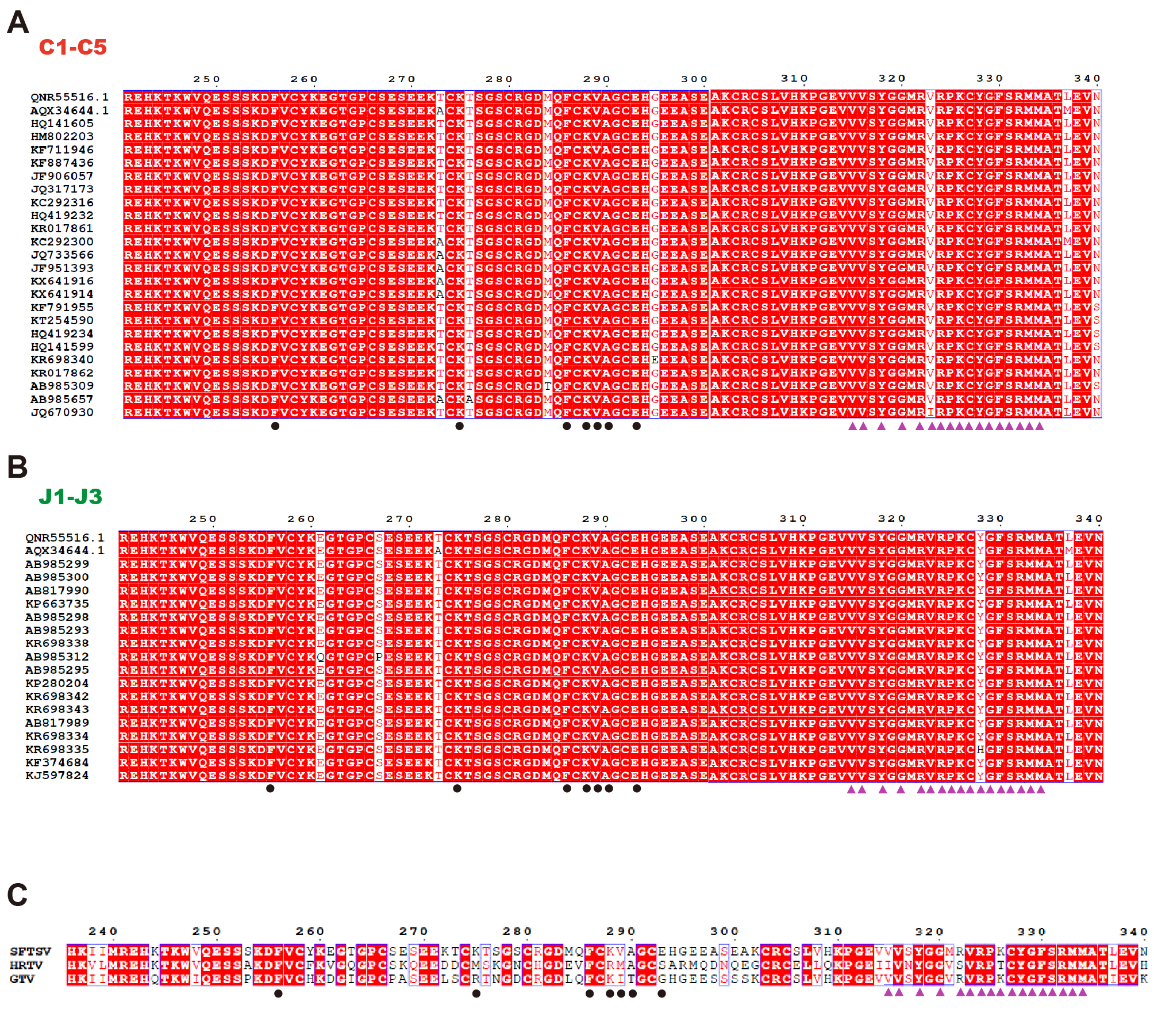

Supplement: S9 Fig — The sequences correspond to those in S2 Fig. The critical binding epitopes of mAb 4–5 are highlighted with black dots, while the binding epitope of Ab10 in the Gn head domain are highlighted with purple triangles. (TIF) [file ppat.1012550.s009.tif]

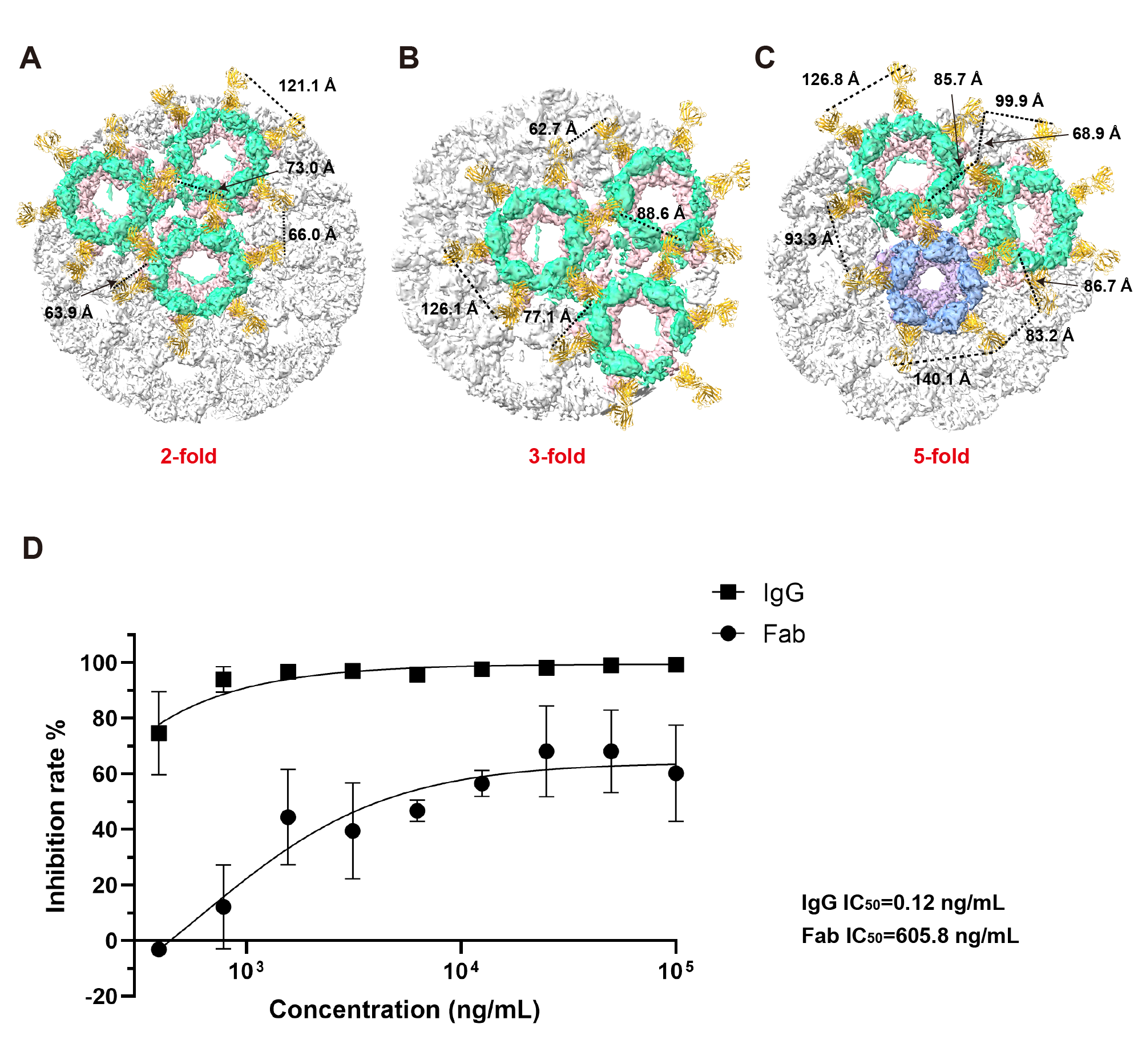

Supplement: S10 Fig — (A, B and C) The distribution and arrangement of mAb 40C10 on different symmetry axes of SFTSV are shown (EMDB: EMD-35176, EMD-35177, EMD-35178; PDB: 8I4T, 7X6W, 7X72). Black dashed lines indicate the distances between the CH1 domain ends of two adjacent mAb 40C10 Fabs, with other colors consistent with those Fig 2. (D) Neutralization profile of mAb 40C10 IgG or Fab to SFTSV. (TIF) [file ppat.1012550.s010.tif]

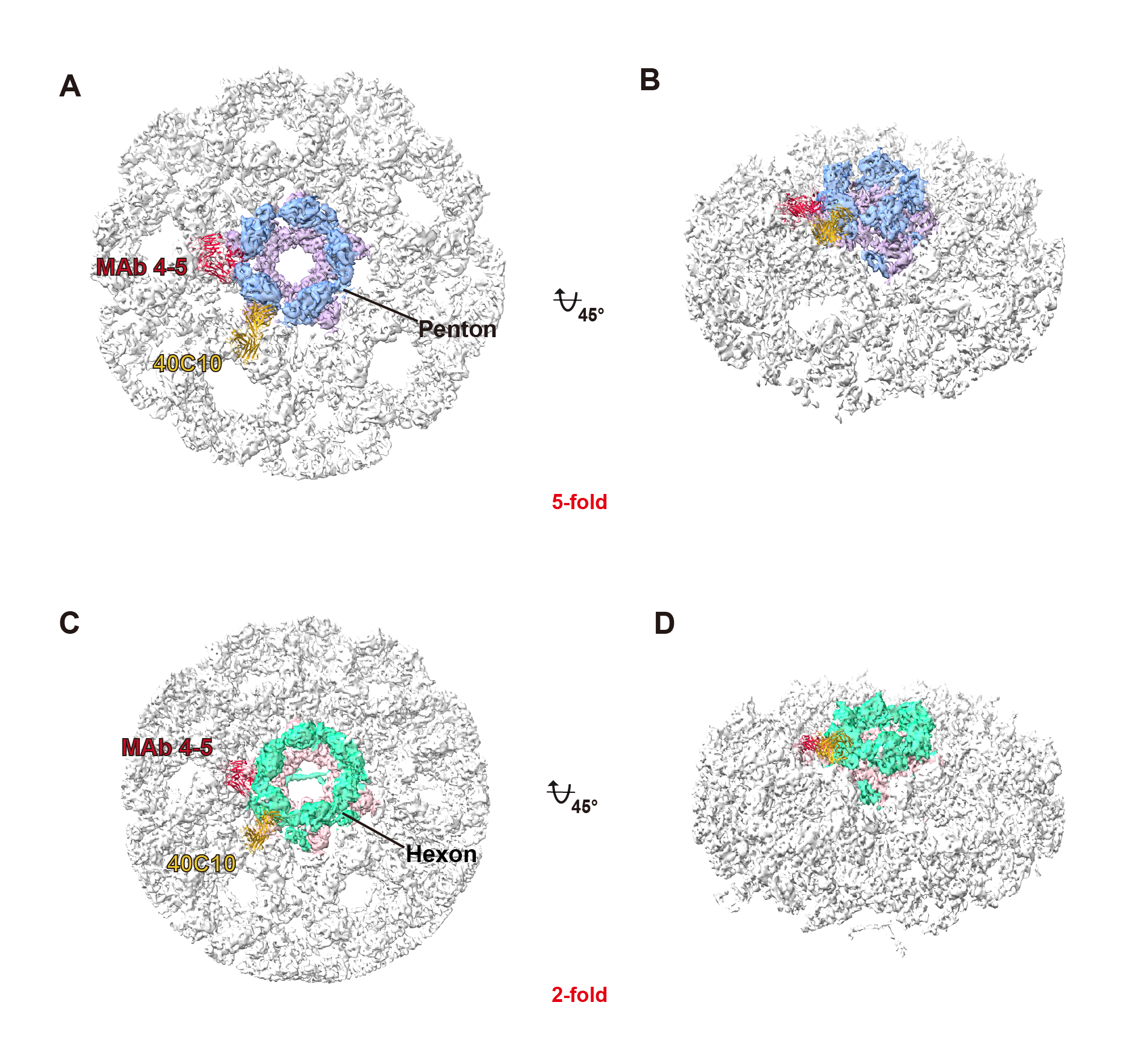

Supplement: S11 Fig — Colors are consistent with those in Fig 2. (EMDB: EMD-35176, EMD-35178; PDB: 8I4T, 7X6W, 7X72). (TIF) [file ppat.1012550.s011.tif]

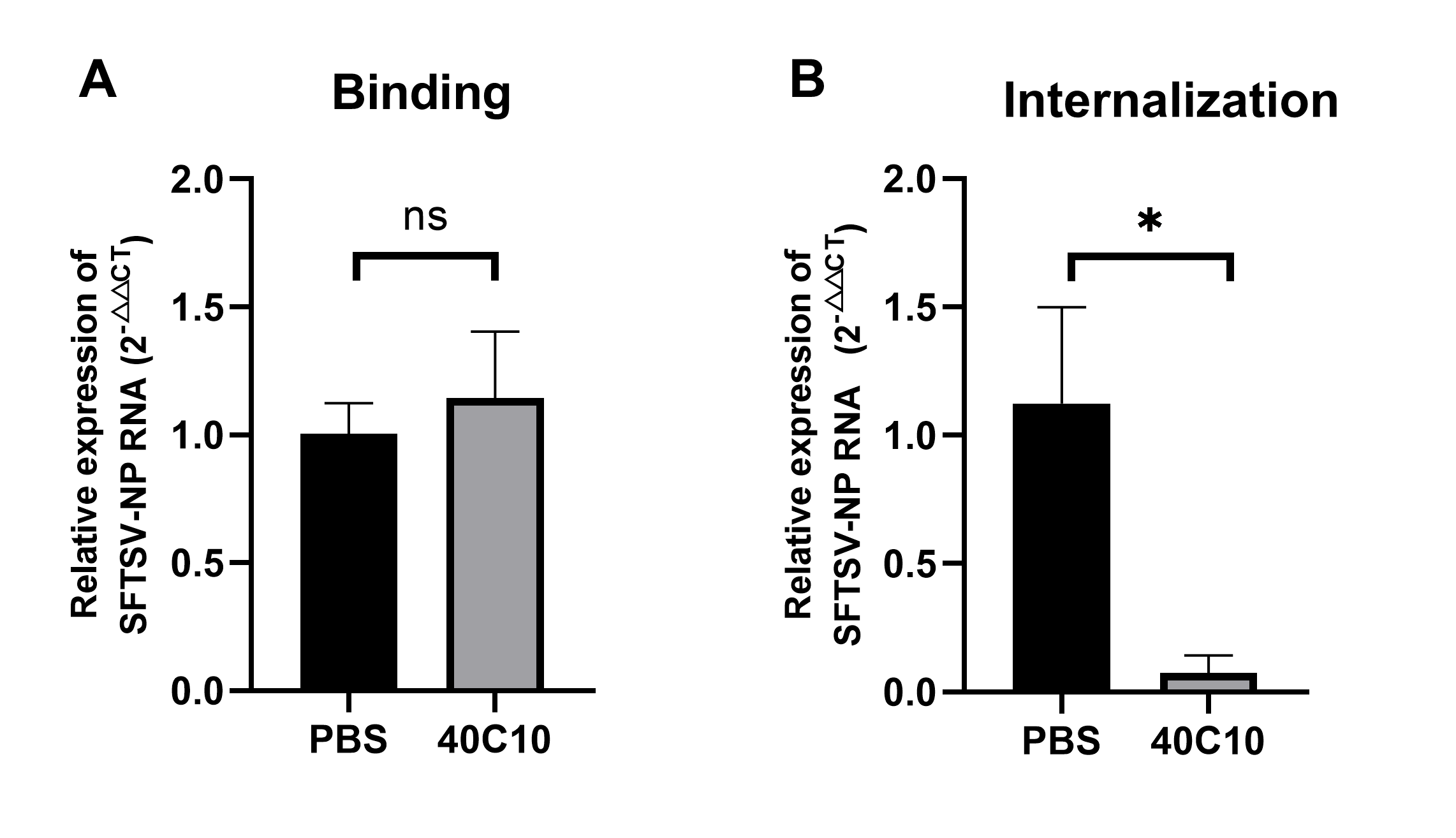

Supplement: S12 Fig — (A) Binding process and (B) Internalization process. (TIF) [file ppat.1012550.s012.tif]

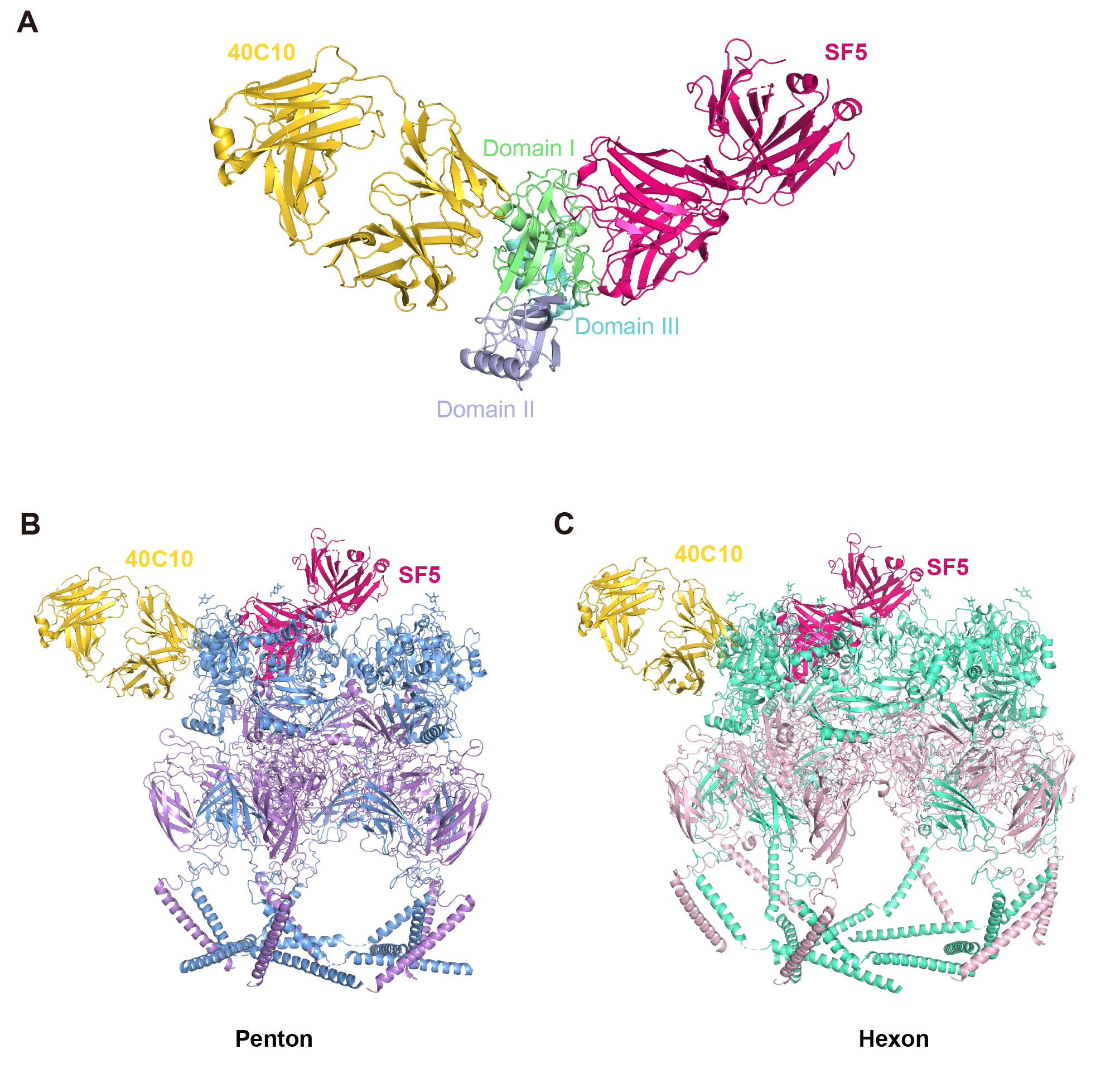

Supplement: S13 Fig — (A) The binding epitopes of mAb 40C10 and SF5 on the SFTSV Gn head are shown. SF5 is colored deep pink, other colors consistent with Fig 2. (B and C) MAb 40C10 and SF5 bind to either the hexon or penton Gn protein. Penton, hexon, mAb 40C10 and SF5 are colored accordingly (PDB: 8I4T, 7X6W, and 7X72). (TIF) [file ppat.1012550.s013.tif]
