# Supplementary material for: Molecular mechanism and structure-guided humanization of a broadly neutralizing antibody against SFTSV
Source: PLoS Pathog. 2024 Sep 25;20(9):e1012550. doi: 10.1371/journal.ppat.1012550 (PMC11423973; doi:10.1371/journal.ppat.1012550)
Supplement: S1 Table — (DOCX) [file ppat.1012550.s014.docx]

**S1 Table. X-ray diffraction data processing and refinement statistics**

|  | SFTSV Gn head–mAb 40C10 complex |
| --- | --- |
| **Data collection** |  |
| Space group | C121 |
| Cell dimensions |  |
| *a, b, c* (Å) | 195.75, 65.91, 73.74 |
| α*, β, γ* (°) | 90.00, 105.38, 90.00 |
| Resolution (Å) | 94.37 - 2.40 (2.46 - 2.40) |
| Unique reflections | 35578 (2622) |
| *I* / *σI* | 4.9 (1.4) |
| Completeness (%) | 99.80 (99.80) |
| R_merge_ | 0.16 (0.90) |
| R_meas_ | 0.22 (0.23) |
| R_pim_ | 0.15 (0.84) |
| CC1/2 | 0.94 (0.62) |
| Mutiplicity | 3.3 (3.2) |
| **Refinement** |  |
| *R*_work_ (%)*^b^* | 20.58 |
| *R*_free_ (%)*^b^* | 24.91 |
| R.m.s. deviations |  |
| Bond lengths (Å) | 0.01 |
| Bond angles (°) | 1.06 |
| Average B factor | 39.37 |
| Ramachandran plot quality |  |
| Most favored region (%) | 95.15 |
| Allowed region (%) | 4.85 |
| Disallowed (%) | 0.00 |

*^a^* Values in parentheses are for highest-resolution shell.

*^b^ R*=Σ*_hkl_* || *F*_obs_ | – *k* | *F*calc | |Σ*_hkl_* | *F_obs_* |, where *R*_free_ is calculated for a randomly chosen 5% of reflections and *R*_work_ is calculated for the remaining 95% of reflections used for structure refinement.
