# Supplementary material for: Molecular mechanism and structure-guided humanization of a broadly neutralizing antibody against SFTSV
Source: PLoS Pathog. 2024 Sep 25;20(9):e1012550. doi: 10.1371/journal.ppat.1012550 (PMC11423973; doi:10.1371/journal.ppat.1012550)
Supplement: S2 Table — (DOCX) [file ppat.1012550.s015.docx]

**S2 Table. PISA analysis of interaction between SFTSV Gn/mAb 40C10**

|  | Total surface area, Å2 | | Interaction residues | | Interface  area Å2 | ΔiG (kcal/mol) | ΔiG  (P-value) | NHB | NSB | NDS | CSS |
| --- | --- | --- | --- | --- | --- | --- | --- | --- | --- | --- | --- |
|  | Gn | HC/LC | Gn | HC/LC |  |  |  |  |  |  |  |
| HC | 14115 | 11411 | 13 | 8 | 262.5 | -0.5 | 0.669 | 4 | 4 | 0 | 0.099 |
| LC |  | 10729 | 18 | 15 | 495.4 | -0.7 | 0.709 | 15 | 2 | 0 | 0.264 |

HC: Heavy chain; LC: Light Chain; ΔiG: Solvation free energy gain upon formation of the interface; N_HB_: number of potential hydrogen bonds across the interface; N_SB_: number of potential salt bridges across the interface; N_DS_: number of potential disulfide bonds across the interface; CSS: Complexation Significance Score.
