# Supplementary material for: Molecular mechanism and structure-guided humanization of a broadly neutralizing antibody against SFTSV
Source: PLoS Pathog. 2024 Sep 25;20(9):e1012550. doi: 10.1371/journal.ppat.1012550 (PMC11423973; doi:10.1371/journal.ppat.1012550)
Supplement: S3 Table — (DOCX) [file ppat.1012550.s016.docx]

**S3 Table. Residues contributed to interaction between SFTSV Gn/mAb 40C10**

| SFTSV Gn | Distance (<4 Å) | mAb 40C10 |
| --- | --- | --- |
| **Hydrogen Bonds** | | |
| ASN 63 [ ND2 ] | 3.09 | VH: TYR 101 [ OH ] |
| LYS 111 [ N ] | 2.87 | VH: TYR 103 [ OH ] |
| ASP 102 [ O ] | 2.99 | VH: ARG 106 [ NH2 ] |
| ASP 102 [ OD1 ] | 2.94 | VH: ARG 106 [ NH1 ] |
| LYS 111 [ NZ ] | 2.87 | VL: ASN 31 [ OD1 ] |
| HIS 64[ O ] | 2.87 | VL: ASN 31[ ND2 ] |
| HIS 64[ ND1] | 2.70 | VL: TYR 50[ OH ] |
| GLN 66[ NE2] | 3.60 | VL: TYR 50[ O ] |
| GLN 66[ NE2] | 2.81 | VL: SER 52[ OG ] |
| GLN 66[ N ] | 2.67 | VL: TYR 53[ OH ] |
| HIS 64[ N ] | 3.81 | VL: TYR 53[ OH ] |
| SER 65[ N ] | 3.40 | VL: TYR 53[ OH ] |
| GLN 66[ NE2 ] | 2.78 | VL: GLY 66[ O ] |
| CYS 156[ N ] | 3.59 | VL: TYR 67[ OH ] |
| LYS 113[ NZ ] | 2.73 | VL: ASN 91[ O ] |
| GLY 114[ N ] | 2.87 | VL: TYR 92[ O ] |
| SER 115[ N ] | 3.54 | VL: ASN 93[ OD1 ] |
| LYS 113[ NZ ] | 3.64 | VL: TYR 96[ OH ] |
| ARG 62[ O ] | 2.53 | VL: TYR 53[ OH ] |
| HIS 64[ O ] | 2.87 | VL: ASN 31[ ND2 ] |
| NAG 1[ O6 ] | 2.65 | VH: ARG 106[ O ] |
| NAG 1[ O7 ] | 2.70 | VH: ARG 106[ NH1 ] |
| **Salt bridges** | | |
| ASP 102 [ OD1 ] | 2.94 | VH: ARG 106 [ NH1 ] |
| ASP 102 [ OD1 ] | 3.26 | VH: ARG 106 [ NH2 ] |
| ASP 102 [ OD2 ] | 3.65 | VH: ARG 106 [ NH1 ] |
| ASP 102 [ OD2 ] | 3.29 | VH: ARG 106 [ NH2 ] |
| LYS 111[ NZ ] | 3.47 | VL: ASP 32[ OD1 ] |
| LYS 111[ NZ ] | 3.91 | VL: ASP 32[ OD2 ] |
