# Supplementary material for: Molecular mechanism and structure-guided humanization of a broadly neutralizing antibody against SFTSV
Source: PLoS Pathog. 2024 Sep 25;20(9):e1012550. doi: 10.1371/journal.ppat.1012550 (PMC11423973; doi:10.1371/journal.ppat.1012550)
Supplement: Attachment — Submitted filename: Cover letter and Response to Reviewer Comments-0817.docx [file ppat.1012550.s017.docx]

Dear Editor,

We greatly appreciate the constructive feedback provided by the reviewers. We are pleased that all three reviewers have recognized the novelty of our work and its suitability for publication in this journal. The manuscript has been thoroughly revised in response to their insightful comments, resulting in significant improvements in quality. In this revised version, all changes to our manuscript have been highlighted within the document using red-colored text. Point-by-point responses to the editor and reviewers are listed below this letter.

Based on the reviewers' suggestions, we designed experiments to preliminarily investigate the neutralization mechanism of the antibody mAb 40C10. Our experimental results show that while mAb 40C10 does not reduce the amount of virus attached to the cells, it does reduce the amount of virus entering the cells. This finding suggests that mAb 40C10 may not prevent the virus from binding to the cells but may affect the virus's internalization process. Additionally, since SFTSV is an icosahedral virus, we explored whether mAb 40C10 might cause intervirion or intravirion cross-links. We fitted the Fab form of mAb 40C10 onto the viral surface at different symmetry axes and measured the distances between the CH1 termini of two adjacent mAb 40C10 Fabs. The results showed that these distances were all greater than the distance between the two Fabs of an IgG molecule. We also measured the neutralization activity of mAb 40C10 in both its IgG and Fab forms, finding that the IgG form had better neutralization activity than the Fab form. Therefore, we suggest that mAb 40C10 may form intervirion cross-links. Based on these observations, we hypothesize that the neutralization mechanism of mAb 40C10 may involve forming cross-links between virions outside the cell, causing them to aggregate. This aggregation could result in larger particle sizes or other effects that hinder viral internalization, thereby preventing the virus from entering and infecting the cells. We have updated the discussion section accordingly.

Additionally, we would like to kindly bring to your attention that we have added a new author, Zidan Song (e-mail: zidan77777@163.com), to the manuscript. We have obtained consent from all other authors regarding this addition and hope it meets with your approval. Zidan Song has made significant contributions to addressing the reviewers' questions and analyzing the crystal structure of the scFv form of HAb-23 in complex with Gn. We sincerely apologize for any inconvenience this addition may cause and appreciate your understanding.

We appreciate the editor's and reviewers' thorough work and hope the corrections meet their approval. Thank you once again for considering our manuscript for publication.

Sincerely,

Nan Zhang, Ph.D.

Nankai University,

Tianjin, China

**Respond to reviewers' comments**

**Original Reviewers comments in black, responses in blue.**

Reviewer Comments (if any, and for reference):

Reviewer's Responses to Questions

**Part I - Summary**
Please use this section to discuss strengths/weaknesses of study, novelty/significance, general execution and scholarship.

Reviewer #1:

In this manuscript, Yang et al. employed X-ray crystallography to determine the crystal structure of the previously studied monoclonal antibody 40C10 in complex with the SFTSV Gn antigen. They analyzed the neutralizing epitope and, based on structural information, performed humanization of the antibody. The affinity and neutralizing activity of the humanized antibodies were subsequently evaluated, and the humanized antibodies retained significant activity. Additionally, the preventive and therapeutic effects of the humanized antibody were evaluated in an animal infection model.

Overall, this manuscript reveals a novel binding epitope through the structural analysis of the complex of antibody 40C10 and SFTSV Gn, elucidating the molecular mechanism of its broad-spectrum neutralizing activity. The epitope was found to be conserved across different genotypes of SFTSV and SFTSV-related viruses. To enhance clinical applicability, the authors humanized of antibody 40C10, and the results showed that the humanized antibodies retained potent neutralizing activity and provided effective protection in an animal infection model, laying a foundation for clinical application. The study is clear and offers new directions for future SFTSV antibody therapies, vaccines, and drug design. The context fits well into the scope of this journal. However, some issues in the manuscript need minor revisions and discussion to enhance the precision of the content.

1. In this manuscript, regarding the structural analysis of the antibody 40C10 and SFTSV Gn, the authors mentioned in line 193 that R106 of HCDR3 forms a hydrogen bond with the glycan molecule of the N-linked glycosylation site N63 of Gn. Is the glycosylation site N63 of Gn conserved across different genotypes of SFTSV? Does this interaction also occur in different genotypes of SFTSV? Interactions between antibodies and glycan molecules of antigens are relatively rare. Therefore, the authors should further analyze in the discussion section whether this interaction is conserved or specific to this particular antibody-antigen pair.

Response:

We thank the reviewer for pointing this out. This is indeed a valuable discussion point. Upon comparing the sequences of different genotypes of SFTSV Gn, we found that the glycosylation site N63 is conserved. Additionally, glycan molecule modification at N63 of SFTSV Gn was observed in the resolved crystal structures of SFTSV Gn and antibody complexes, as well as in the cryo-EM structure of the SFTSV virion. Therefore, the hydrogen bond interaction between mAb 40C10 and the glycan molecule of the N-linker glycosylation site N63 is conserved across different genotypes in the antigen-antibody interaction. We have updated the discussion as follows **(Lines 352–358)**:

"Besides, hydrogen bond interaction between mAb 40C10 and the glycan molecules of the N-linked glycosylation site N63 of Gn was observed. This glycosylation site is conserved across different genotypes of SFTSV (S2A and S2B Fig), and glycan modification at this site has been observed in several resolved structure of SFTSV Gn [11, 17]. These observations suggest that the interaction between the antibody and SFTSV Gn is conserved and that glycosylation at this site may play a critical role in antibody binding."

2. The authors previously found that the antibody 40C10 exhibited cross-neutralizing activity against HRTV and GTV and analyzed the potential molecular mechanisms underlying this cross-reactivity through structural and sequence analyses. However, they did not experimentally verify whether the humanized antibody HAb-23 retains this cross-neutralizing activity. Conducting experiments to validate that the humanized antibody still possesses cross-neutralizing activity would strengthen the conclusions of the study.

Response:

We thank the reviewer for this suggestion. We strongly agree with the reviewer's opinion that further experimental validation of the cross-neutralizing activity of the humanized antibody HAb-23 against HRTV and GTV is crucial. To address this, we initially aimed to investigate whether the humanized antibody HAb-23 maintains structural and interaction similarities with the mouse antibody mAb 40C10 by resolving the complex structure of HAb-23 with Gn. We successfully obtained the crystal structure of the scFv form of HAb-23 in complex with Gn, but the resolution was relatively low at 3.75 Å. The R_work_ and R_free_ values of the complex structure were 0.2724 and 0.3081, respectively **(R-Fig A)**. Structural comparison revealed an RMSD of between this complex and the mAb 40C10-Gn complex was 0.932, and the RMSD of between HAb-23 and mAb 40C10 was 0.955 **(R-Fig B)**. The CDR regions of HAb-23 that interact with Gn are identical to those in mAb 40C10, with a few mutations in the framework regions. These mutations did not affect the structure or binding to Gn. Based on these observations, we believe that HAb-23 retains the neutralizing activity of mAb 40C10, including its cross-neutralizing activity against HRTV and GTV. Due to the lower resolution of the data, we did not include these results in the main manuscript.

We apologize for not completing the experiment to confirm HAb-23's cross-neutralizing activity against HRTV and GTV within the limited time. However, the structural data we present supports the conclusion that HAb-23 maintains this cross-neutralizing activity.

**R-Fig. Structure analysis of the SFTSV Gn head–HAb-23 scFv complex.** (A) The crystal structure of the SFTSV Gn head–HAb-23 scFv complex. The SFTSV Gn head is colored paleyellow and palecyan. The HAb-23 heavy chain and light chain are colored lightpink and orange, slate and splitpea, respectively. (B) Structural comparison between the SFTSV Gn head–HAb-23 scFv complex and the SFTSV Gn head–mAb 40C10 Fab complex. The SFTSV Gn head–mAb 40C10 Fab complex is colored gray, the SFTSV Gn head–HAb-23 scFv complex is colored as described above.

3. In Fig 3D, the authors used log2 for the x-axis to represent antibody concentration. However, antibody concentration should typically be expressed in log10 rather than log2, as this facilitates easier interpretation of the results.

Response:

We appreciate the reviewer's careful attention to the figure details in our manuscript. We have modified **Fig 3D** according the reviewer's opinion.

**Fig 3. Evaluation of humanized antibodies.**

4. In the animal experiment described in line 302, the authors used a dose of 50 LD50 for infecting mice. Why was the infection dose chosen as LD50 instead of using TCID50 or PFU?

Response:

We appreciate the reviewer's comment and the opportunity to clarify our rationale. In our animal experiments, we observed inconsistent mortality of SFTSV strains at the same TCID50 dose in our animal experiment as previously reported [1]. Therefore, selecting the LD50 as the infection dose helps eliminate the variability in virulence among different strains. Additionally, to maintain consistency with the previous study of 40C10 [1], we chose the LD50 as the infection dose.

5. Recently, a paper titled "Bispecific antibodies targeting two glycoproteins on SFTSV exhibit synergistic neutralization and protection in a mouse model" was published in PNAS. In this study, several antibodies were isolated from an SFTS convalescent patient, and structural analysis revealed that one of these antibodies, SF5, binds to subdomain I of the SFTSV Gn. However, upon comparison, it was found that the specific epitope of SF5 differs from the antibody 40C10 reported in this manuscript. It is recommended to update the discussion section with this latest research. A detailed comparison and discussion of both the structural and animal experiment results should be included.

Response:

We thank the reviewer for the constructive advice. We thoroughly reviewed the literature cited by the reviewer and compared mAb 40C10 with SF5 mentioned in the paper [2]. We have updated the discussion of our manuscript to include these comparisons **(Lines 455–469)**:

"While the manuscript was under peer-review, Chang et al. reported several neutralizing antibodies targeting SFTSV glycoproteins, including one that interact with domain I of SFTSV Gn [43]. Structural comparison clearly shows that mAb 40C10 and SF5 recognize different epitopes on domain I, with no overlap between the two (S13A Fig). The affinity and neutralizing activity of mAb 40C10 against Gn surpass those of SF5. This may be because the epitope of SF5 is hidden within the penton/hexon, while the epitope of mAb 40C10 is located on the external surface of the penton/hexon, allowing mAb 40C10 to bind more effectively to each Gn in the penton/hexon (S13B and S13C Fig). In animal experiment, Chang et al. used a challenge dose of 50,000 LD50 and tested the protective effects of different antibody doses. A dose of 5 mg/kg of SF5 achieved 100% prophylactic protection, and a dose of 10 mg/kg achieved 100% therapeutic efficacy. Unfortunately, we did not experiment with different doses of mAb 40C10 to determine the minimum dose required for effective prophylactic and therapeutic effects. This aspect will be addressed in future studies to better understand the optimal dosing strategy."

**S13 Fig. Analysis of binding epitopes of mAb 40C10 and SF5.** (A) The binding epitopes of mAb 40C10 and SF5 on the SFTSV Gn head are shown. SF5 is colored deep pink, other colors consistent with Fig 2. (B and C) MAb 40C10 and SF5 bind to either the hexon or penton Gn protein. Penton, hexon, mAb 40C10 and SF5 are colored accordingly (PDB: 8I4T, 7X6W, and 7X72).

6. There are also formatting issues in the manuscript that need attention: For instance, in line 205, the sentence “… exhibited a 3-5-fold decrease…” should use an en dash (–) to represent "to", thus it should be written as "3–5." Similarly, in lines 88-89 and line 201, the sentence "C1-C5" and "J1-J3" should also use an en dash, becoming "C1–C5" and "J1–J3," respectively.

Additionally, in line 306, the legend of Fig 4 needs to correctly format the mouse type symbols.

Response:

We apologize for the error and any confusion caused. These have been corrected in the revised vision. Thank you for bringing these to our attention, and we appreciate your careful review and valuable feedback.

Reviewer #2:

The manuscript titled “Molecular mechanism and structure-guided humanization of broadly neutralizing antibody against SFTSV” by Pinyi Yang and colleagues performed structural studies on a previously reported monoclonal antibody called 40C10 against the Severe fever with thrombocytopenia syndrome virus. The crystal structure of antibody 40C10 in complex with SFTSV glycoprotein N revealed a novel antibody targeting site on Gn that has not been reported previously. This epitope, located on domain I of Gn head, is highly conserved among subtypes, explaining the broad neutralization by 40C10. The authors then performed humanization on this antibody and obtained 3 heavy and 3 light chains, respectively. They expressed 9 antibodies by combinations of new heavy and light chains and performed binding and neutralizations and identified one antibody to test its prophylactic and therapeutic potentials. Overall, the authors identified a new antibody targeting site on Gn and obtained humanized version of this mouse antibody.

Reviewer #3:

The authors describe the structural basis of epitope recognition of a potently neutralizing murine antibody 40C10 on SFTSV. The epitope is on the Gn glycoprotein domain I of its head region. The authors also provide evidence of the potential therapeutic applicability of the humanized version of the mAb. Taken together, this work adds to the growing list of anti-viral antibodies currently under development against many different viruses and is of broad interest to the audience of PLOS Pathogens.

**Part II – Major Issues: Key Experiments Required for Acceptance**
Please use this section to detail the key new experiments or modifications of existing experiments that should be absolutely required to validate study conclusions.

Generally, there should be no more than 3 such required experiments or major modifications for a "Major Revision" recommendation. If more than 3 experiments are necessary to validate the study conclusions, then you are encouraged to recommend "Reject".

Reviewer #1: see the report.

Reviewer #2: None

Reviewer #3: The authors describe the structural basis of epitope recognition of the murine antibody 40C10 on the head portion of the viral glycoprotein Gn using X-ray crystallography. Additionally, they produce humanized version of this mAb which maintain potent neutralization capability in vitro as well as in vivo.

However, there are a few aspects that if addressed can enrich this manuscript significantly.

1. The authors fit the crystal structures of the Fab + Gn head domain onto the existing model of the STSFV determined by Cryo-EM. They show that the Fab does not have any major clashes at either on the hexon or penton positions and therefore, in principle can saturate all the binding sites. Could the authors suggest whether the mAb 40C10 can form intra-/inter-virion crosslinks on the viral surface based on the distance criteria between the Fab arms of an IgG? The authors should check the following paper from 2023: DOI: 10.1073/pnas.2213690120. The paper delves into the possible link between neutralization potencies of neutralizing mAbs and their arrangement on icosahedral viruses. The authors should also do the same analysis with the other mAb (mAb 4-5) for which a structural model exists. It would be interesting to see if both mAbs 40C10 and 4-5 can form intra-virion crosslink or not. The reason I am suggesting this is because SFTSV is icosahedral.

Response:

We appreciate the reviewer's insight and the opportunity to address this important point. We have thoroughly reviewed the literature cited by the reviewer [3]. Additionally, we have examined related studies to provide new insights into the neutralization mechanism of mAb 40C10. We fitted mAb 40C10 to different symmetry axes of the SFTSV, observed the arrangement of mAb 40C10 on the icosahedral structure of SFTSV, and measured the distances between the CH1 termini of adjacent mAb 40C10 Fabs. These distances ranged from 62.7 Å to 140.1 Å, all greater than the distance between two Fabs of an IgG molecule [4]. Therefore, we suggest that mAb 40C10 might form intervirion cross-links. To further confirm this potential mechanism, we purified mAb 40C10, enzymatically cleaved it to obtain the Fab form, and measured the IC50 values of mAb 40C10 in both its IgG and Fab forms **(S10 Fig)**. The results showed that the IgG form had a superior neutralization effect compared to the Fab form, supporting the hypothesis that mAb 40C10 neutralizes SFTSV through intervirion cross-linking. Additionally, we fitted mAb 4-5 and found that its epitope is relatively hidden. Before the conformational changes in SFTSV glycoproteins, it is unlikely to form intravirion or intervirion cross-links. This content has been updated in the discussion section **(Lines 388–406)**:

"Neutralizing antibodies against enveloped virus can exert their effects through multiple mechanisms [36]. Studies have shown that by analyzing the cryo-EM structures of three anti-Eastern equine encephalitis virus (EEEV) neutralizing human mAbs bound to EEEV, the distance between Fab regions bound to glycoprotein spikes can elucidate the mechanism of neutralization through intravirion or intervirion cross-links [37]. Similarly, mAb 40C10 may neutralize SFTSV, an icosahedral virus, through a mechanism involving virion cross-linking. We fitted mAb 40C10 to the surface of SFTSV at the 2-fold, 3-fold, and 5-fold symmetry axes and measured the distances between the CH1 domain ends of two adjacent mAb 40C10 Fabs. The distances ranged from 62.7 Å to 140.1 Å, all of which are greater than the distance between the two Fabs of an IgG molecule [38] (S10A, S10B and S10C Fig). This observation suggests that mAb 40C10 might form intervirion cross-links. To further substantiate this hypothesis, we measured the IC_50_ values of mAb 40C10 in both its IgG and Fab forms (S10D Fig). The significantly better neutralization effect observed with the IgG form compared to the Fab form supports the hypothesis that the potential neutralization mechanism of mAb 40C10 involves intervirion cross-linking. Furthermore, we also fitted mAb 4-5 onto the SFTSV surface and found that its epitope is located internally rather than on the surface (S11 Fig). Thus, mAb 4-5 may not bind to Gn or form virion cross-links before the conformational changes in SFTSV glycoproteins."

We once again thank the reviewer for their constructive comment, which has helped us analyze the neutralization mechanism of mAb 40C10 from a different perspective, enriching the discussion in our manuscript.

**S10 Fig. MAb 40C10 neutralizes SFTSV by forming intervirion cross-links.** (A, B and C) The distribution and arrangement of mAb 40C10 on different symmetry axes of SFTSV are shown (EMDB: EMD-35176, EMD-35177, EMD-35178; PDB: 8I4T, 7X6W, 7X72). Black dashed lines indicate the distances between the CH1 domain ends of two adjacent mAb 40C10 Fabs, with other colors consistent with those Fig 2. (D) Neutralization profile of mAb 40C10 IgG or Fab to SFTSV.

2. The epitope recognized by 40C10 is conserved among different strains of SFTSV as well as among HRTV and GTV. The Multiple sequence alignment in Fig S2 shows significant similarity among the different strains. Could the authors also show the residues that constitute the footprint for mAb 4-5? If so, then it should also exhibit broad neutralizing capability against different strains. The authors should discuss at least a bit about the extent of sequence conservation of the three different epitopes targeted by the three antibodies, mAb 40C10, mAb 4-5 and Ab10.

Response:

We appreciate the reviewer's insightful comment. In response to this feedback, we have conducted a sequence conservation analysis of the binding epitopes of MAb 4-5 and Ab10 across different genotypes of SFTSV and SFTSV-related virus. This analysis has been updated in our manuscript **(Lines 359–373)**:

"Sequence alignment of different SFTSV genotypes shows that the binding epitope of mAb 40C10 is conserved, which is consistent with its effective neutralizing effect against various SFTSV genotypes in vitro [20]. Similarly, the critical binding epitopes of mAb 4-5 and Ab10 in the Gn head domain are conserved across different SFTSV genotypes (S9A and S9B Fig) [17, 18], suggesting that mAb 4-5 and Ab10 might also broadly neutralize different SFTSV genotypes. Experimental data indicate that mAb 40C10 can cross-neutralize HRTV and GTV, with sequence analysis revealing that its binding epitope is relatively conserved in these SFTSV-related viruses. However, the critical binding epitopes of mAb 4-5 show lower conservation in HRTV and GTV (S9C Fig), indicating potentially weaker neutralization activity against these viruses. In contrast, the binding epitopes of Ab10 in the Gn head domain is relatively conserved in HRTV and GTV (S9C Fig), suggesting possible neutralization of these viruses. Further experimental validation is needed to confirm whether mAb 4-5 and Ab10 possess broad-spectrum neutralizing activity against different genotypes of SFTSV and SFTSV-related viruses."

**S9 Fig. Sequence alignment among various genotypes of SFTSV and other strains.** The sequences correspond to those in S2 Fig. The critical binding epitopes of mAb 4-5 are highlighted with black dots, while the binding epitope of Ab10 in the Gn head domain are highlighted with purple triangles.

3. What is the mechanism of action of this antibody? Does it block attachment or receptor engagement or fusion? there is some discussion about this, but no experimental evidence. It would really enrich the manuscript if the authors can show if the antibody blocks either attachment or receptor engagement or fusion or all of them. If it is currently beyond the scope of the study, then if they could show that the antibody is able to block before or after attachment to cells, then that would be helpful too.

Response:

We thank the reviewer for the constructive advice. Based on your suggestions, we conducted experiments to investigate the mechanism of action of the monoclonal antibody mAb 40C10.

First, to determine whether mAb 40C10 affects virus attachment to cells, we incubated the virus with either mAb 40C10 or PBS, added the mixture to the cells, placed it at 4°C for 1 hour, and then washed to remove unbound virus. We extracted RNA and used qRT-PCR to detect the SFTSV-NP nucleic acid and GAPDH. The results showed that no significant difference in the amount of virus adsorbed to cells between the PBS group and the mAb 40C10 group **(S12A Fig)**.

Previous studies have shown that SFTSV enters the cells through endocytosis into the cytoplasm, and then undergoes membrane fusion with the late endosome membrane in the acidic environment, releasing the virus to complete the invasion [5]. Therefore, we further explored whether mAb 40C10 would affect the virus endocytosis based on the characteristics of SFTSV invasion of cells. We repeated the virus adsorption steps, then cultured the cells in a medium containing NH_4_Cl at 37°C for 1 hour, which inhibits the fusion of the virus with the endosome membrane [6]. After washing to remove the virus that did not enter the cells, we extracted RNA and used qRT-PCR to detect the SFTSV-NP nucleic acid and GAPDH. The results showed that the amount of virus entering the cells in the mAb 40C10 group was significantly lower than that in the PBS group, suggesting that the antibody affected the internalization process of SFTSV **(S12B Fig)**.

In summary, while we were unable to fully elucidate mAb 40C10's neutralization mechanism within the time constraints, our results suggest that mAb 40C10 does not block the attachment of SFTSV to host cells but likely inhibits the virus's internalization process. We apologize for the incomplete exploration of the neutralization mechanism, but the current results provide valuable insights. We plan to conduct further studies to investigate this mechanism more comprehensively in the future.

The above content has been updated in the discussion section (**Lines 407–426**), and the corresponding experimental methods have been added to the materials and methods.

**S12 Fig. MAb 40C10 inhibition effects on the SFTSV infection process.** (A) Binding process and (B) Internalization process.

If these points are addressed, then I am happy to recommend the paper for publication in PLOS Pathogens.

**Part III – Minor Issues: Editorial and Data Presentation Modifications**
Please use this section for editorial suggestions as well as relatively minor modifications of existing data that would enhance clarity.

Reviewer #1: see the report.

Reviewer #2: 1. Docking the 40C10 structure on virion structure indicated the Fab binding is compatible with both penton and hexon, can the authors show RMSD on alignment of Gn in crystal structure and in virion? Can the authors discuss what is the potential mechanism of 40C10 against SFTSV infection?

Response:

We appreciate the reviewer's valuable suggestions. We have aligned the Gn in the crystal structure with the Gn in the cryo-EM structure of the SFTSV virion, and the RMSD values are consistently below 2 Å **(S4 Fig)**. This indicates a high degree of structural similarity between the two structures. We had included this information in the revised manuscript for clarity **(Lines 237–240)**.

**S4 Fig. Structural comparison between the Gn in the SFTSV Gn head–mAb 40C10 complex crystal structure and the Gn in the cryo-EM structure of the SFTSV virion.** (A and B) The Gn in the SFTSV Gn head–mAb 40C10 complex crystal structure is shown in gray. (A) The Gn-Gc of SFTSV 2-fold hexamer (PDB: 7X6W) is displayed in color. (B) The Gn-Gc of SFTSV 5-fold pentamer (PDB: 7X72) is shown in color.

In response to your suggestions about the neutralization mechanism of mAb 40C10, we have conducted additional experiments and have updated the discussion section to include these findings **(Lines 407–426)**:

"To further understand the neutralizing mechanism of mAb 40C10, we first explored whether mAb 40C10 affects the virus attachment to cells. Experimental results showed that the amount of virus adsorbed to the cells in the PBS group and the mAb 40C10 group was similar, with no significant difference (S12A Fig). Previous studies have demonstrated that SFTSV enters cells through endocytosis into the cytoplasm, followed by membrane fusion with the late endosome membrane in the acidic environment, which releases the virus to complete the invasion process [39]. Based on these characteristics of SFTSV invasion of cells, we investigated whether the antibody affects virus endocytosis. During the early stage of virus adsorption, consistent with the previous steps, we added NH_4_Cl to the cell culture medium, which can inhibit the fusion of the virus with the endosome membrane [40]. The experimental results showed that the amount of virus entering the cells in the mAb 40C10 group was significantly lower than that in the PBS group (S12B Fig), suggesting that the antibody affected the internalization process of SFTSV. These observations, combined with the data suggesting that mAb 40C10 might form intervirion cross-links, lead us to hypothesize that mAb 40C10 may not interfere with the binding of the virus to cell receptors. Instead, it may form cross-links between virions outside the cell, causing them to aggregate. This aggregation could result in larger particle sizes or other effects that hinder viral internalization, thereby preventing the virus from entering and infecting the cells. However, this hypothesis requires further experimental validation."

2. Is it possible to show in Figure 1 as another panel in which the Gn is shown in surface representation and colored by sequence variation with the footprint of 40C10 highlighted marked?

Response:

We sincerely appreciate the reviewer's suggestion. In response to this feedback, we have updated a figure to clearly illustrate the binding epitope of mAb 40C10. The surface of domain I of Gn has been colored based on sequence variation. In the main text, the "Analysis of the binding epitope of mAb 40C10 on SFTSV Gn" section includes a conservation analysis of the epitope of mAb 40C10 across different genotypes of SFTSV. To provide a clearer explanation and presentation, we have included this figure in the supporting information as **S3 Fig**. We hope this meets the reviewer's expectations.

**S3 Fig. Overall structure of the SFTSV Gn head–mAb 40C10 complex.** (A and B) The SFTSV Gn head is shown as surface. Domain I of the SFTSV Gn head is colored by sequence variation using the ConSurf server [7, 8], while domains II and III are colored gray. The mAb 40C10 heavy and light chains are colored salmon and yellow, respectively. (B) The binding region of mAb 40C10 on the SFTSV Gn head is indicated. The CDR loops of mAb 40C10 colored as described above.

3. The authors claimed they have "humanized" this antibody based on a website, it will be helpful to show the humanness score of the mouse version as a "negative control" and may use another know human antibody as a "positive control".

Response:

We appreciate the reviewer's insightful comment. We agree that providing a humanness score for the mouse antibody as a negative control, along with a known human antibody as a positive control, would strengthen our analysis. We have included these comparisons in our revised manuscript. The humanness scores for the heavy and light chains of mouse antibody (mAb 40C10) used as negative controls, are less than 0.025. In contrast, the human antibody (SARS-CoV-2 neutralizing antibody TH132, PDB: 7YVG [9]) used as a positive control, has a humanness score of 1. This content has been added to the supporting information **S6 Fig**.

**S6 Fig. Humanness score of the heavy and light chains of human antibody/mouse antibody.** (A) The human antibody (SARS-CoV-2 neutralizing antibody TH132) [9] and the mouse antibody (mAb 40C10) were scored for their degree of humanization using the “Hu-mAb” website. The human antibody serves as a positive control, while the mouse antibody serves as a negative control for humanization scoring.

4. The author did not provide any experimental data on the "humanness", for example, the pharmacokinetics comparison in a model system, such as the Human FcRn transgenic mice.

Response:

We sincerely appreciate the reviewer's important and insightful comment. Since SFTSV strains are usually only lethal in animal models with immunodeficiency, such as IFNAR^-/-^ C57BL/6 mice [10]. We have previously considered this approach and attempted to use the Human FcRn transgenic mice model. However, we encountered challenges with the stability of SFTSV infection in this model.

We acknowledge the importance of this data and will continue our efforts to optimize this model in future research. This will enable us to provide more comprehensive data on the pharmacokinetics and humanness of our antibody. Thank you once again for your valuable suggestion.

5. Line 233 typo "exit" should be "exist".

Response:

We apologize for the typographical error. We have corrected "exit" to "exist" in the revised manuscript. Thank you for pointing this out.

Reviewer #3: There are some grammatical mistakes. Please do a word check.

Response:

We apologize for the grammatical mistakes. We have carefully reviewed and corrected all identified mistakes in the revised version.

**References**

1.	Wu X, Moming A, Zhang Y, Wang Z, Zhang T, Fu L, et al. Identification and characterization of three monoclonal antibodies targeting the SFTSV glycoprotein and displaying a broad spectrum recognition of SFTSV-related viruses. PLoS Negl Trop Dis. 2024;18(6):e0012216. pmid: 38848311.

2.	Chang Z, Gao D, Liao L, Sun J, Zhang G, Zhang X, et al. Bispecific antibodies targeting two glycoproteins on SFTSV exhibit synergistic neutralization and protection in a mouse model. Proc Natl Acad Sci U S A. 2024;121(24):e2400163121. pmid: 38830098.

3.	Williamson LE, Bandyopadhyay A, Bailey K, Sirohi D, Klose T, Julander JG, et al. Structural constraints link differences in neutralization potency of human anti-Eastern equine encephalitis virus monoclonal antibodies. Proc Natl Acad Sci U S A. 2023;120(13):e2213690120. pmid: 36961925.

4.	Sharma A, Zhang X, Dejnirattisai W, Dai X, Gong D, Wongwiwat W, et al. The epitope arrangement on flavivirus particles contributes to Mab C10's extraordinary neutralization breadth across Zika and dengue viruses. Cell. 2021;184(25):6052-66 e18. pmid: 34852239.

5.	Liu J, Xu M, Tang B, Hu L, Deng F, Wang H, et al. Single-Particle Tracking Reveals the Sequential Entry Process of the Bunyavirus Severe Fever with Thrombocytopenia Syndrome Virus. Small. 2019;15(6):e1803788. pmid: 30589216.

6.	Shen S, Zhang Y, Yin Z, Zhu Q, Zhang J, Wang T, et al. Antiviral activity and mechanism of the antifungal drug, anidulafungin, suggesting its potential to promote treatment of viral diseases. BMC Med. 2022;20(1):359. pmid: 36266654.

7.	Ashkenazy H, Abadi S, Martz E, Chay O, Mayrose I, Pupko T, et al. ConSurf 2016: an improved methodology to estimate and visualize evolutionary conservation in macromolecules. Nucleic Acids Res. 2016;44(W1):W344-50. pmid: 27166375.

8.	Yariv B, Yariv E, Kessel A, Masrati G, Chorin AB, Martz E, et al. Using evolutionary data to make sense of macromolecules with a "face-lifted" ConSurf. Protein Sci. 2023;32(3):e4582. pmid: 36718848.

9.	Guo Y, Zhang G, Yang Q, Xie X, Lu Y, Cheng X, et al. Discovery and characterization of potent pan-variant SARS-CoV-2 neutralizing antibodies from individuals with Omicron breakthrough infection. Nat Commun. 2023;14(1):3537. pmid: 37322000.

10.	Matsuno K, Orba Y, Maede-White K, Scott D, Feldmann F, Liang M, et al. Animal Models of Emerging Tick-Borne Phleboviruses: Determining Target Cells in a Lethal Model of SFTSV Infection. Front Microbiol. 2017;8:104. pmid: 28194148.
